# Effects of rumen-protected guanidinoacetic acid on fat deposition, antioxidant capacity, fatty acid profiles in subcutaneous adipose of Tibetan sheep: integrated RNA-seq and LC-MS/MS analysis

**DOI:** 10.3389/fvets.2026.1866223

**Published:** 2026-08-25

**Authors:** Zhenglu Yang, Chengdi Shi, Zhendong Liu, Rengeerli Sa, Yu Zhang, Qiurong Ji, Lijuan Han, Xiaohong Wang, Shengzhen Hou, Linsheng Gui

**Affiliations:** 1 College of Agriculture and Animal Husbandry, Qinghai University, Xining, China; 2 Maduo County Agriculture and Animal Husbandry Technology and Rural Revitalization Comprehensive Service Center, Qinghai, China; 3 Guinan County Animal Husbandry and Veterinary Station, Qinghai, China

**Keywords:** GAA, LC-MS/MS, RNA-Seq, subcutaneous adipose tissue, Tibetan sheep

## Abstract

**Introduction:**

The subcutaneous fat exerts energy storage and protective effects. However, excessive fat deposition results in waste of biological resources and compromises meat quality. According to the biological function, we hypothesized that GAA's could reduce subcutaneous fat deposition in Tibetan sheep.

**Methods:**

A total of 120 healthy weaned male lambs (2 months old) with similar body weight (17.33 ± 0.21 kg) were randomly divided into four groups, each receiving a basal diet or diet containing 0.08%, 0.10%, or 0.12% GAA. The experimental period lasted 90 days.

**Results:**

Our results showed that 0.10% GAA significantly reduced subcutaneous fat thickness (*P* < 0.05). Antioxidant capacity was improved, as evidenced by a reduced MDA (*P, P*-linear, *P*-quadratic < 0.05), while increased SOD and T-AOC (*P, P*-linear, *P*-quadratic < 0.05). The contents of C18:3(n-3) (*P*, P-quadratic < 0.05) and C18:2(n-6) (*P, P*-linear < 0.05) was significantly increased when supplementing 0.10% GAA, while those of C16:0 (*P* < 0.05) and C22:2(n-6) (*P, P*-linear < 0.05) was significantly decreased. Pearson correlation analysis revealed that the differential genes including *ANGPT2, PLA2G4A, PIK3CB, IDNK, MAPKAPK5* and the differential metabolites including DG (16:0/20:4), TG (16:0/16:0/18:1), DG (16:1/18:1), DG (16:0/18:2), Cer (d18:1/18:1) were closely associated with the antioxidant capacity and fat deposition.

**Conclusion:**

In summary, 0.10% GAA reduced subcutaneous fat thickness, improved the fatty acid profile, and maintained redox homeostasis in Tibetan sheep.

## 1 Introduction

As a naturally occurring compound, creatine is synthesized endogenously from arginine, glycine and methionine (1). Via regulating phosphagen system, creatine serves as a critical energy buffer for cellular processes (2). However, considering its relative instability and expensive costs, creatine was universal replaced by its metabolic precursor GAA in the animal nutrition industry. Dietary supplementation with GAA promoted the portal nutrient concentrations and hepatic IGF-1 expression, thereby contributing to the growth performance in Hu sheep (3). Similarly, dietary varying levels of GAA (1 g/100 kg BW, and 2 g/100 kg BW) promoted IGF-1 and MHC IIA, and then affected the skeletal muscle growth in finishing beef steers (4). In ovarian tissue, GAA influenced the follicular growth and estrogen synthesis through inhibiting the CYP19A1 and AKT1 transcript levels in multiparous sheep (5). Furthermore, the addition of 0.11% GAA in diet improved average daily gain in yak, which associated with arginine and tryptophan metabolism (6).

Currently, growing research demonstrate that GAA was involved in the glycolipid metabolism by modulating mRNA or non-coding RNA expression. Dietary supplementation of GAA decreased the expression of mitochondrial-related genes, thereby inhibiting inguinal white adipose tissue in high-fat diet (HFD)-fed mice. This process supports ameliorated hepatic steatosis and lipid deposition (7). A total of 149 serum difference metabolites were identified when supplemented GAA in ducks, which mostly enriched in glycolipid metabolism (i. e., fatty acid degradation, fat digestion and absorption, and lipid accumulation) (8). GAA supplementation reduced fat deposition and promoted lipid oxidation by regulating miR-103/107–SCD/TMEM35B axis in broiler chickens (9).

The subcutaneous fat exerts energy storage and protective effects. However, excessive fat deposition results in waste of biological resources and compromises meat quality (10). According to the biological function, we hypothesized that GAA's could reduce subcutaneous fat deposition in Tibetan sheep. Therefore, the study systematically evaluated the histomorphological structure, antioxidant capacity, and fatty acid content of subcutaneous adipose tissue. In addition, RNA-seq and LC-MS/MS technologies were integrated to explore the effects of GAA on subcutaneous fat-related genes and metabolites in Tibetan sheep, thereby providing data support for the application of GAA in the nutritional regulation of Tibetan sheep.

## 2 Materials and methods

### 2.1 Ethics statement

All experimental animal procedures complied with the laboratory animal management and welfare regulations approved by the Ethics Committee of Qinghai University, Xining, Qinghai, China. All animal experiments were approved by the Animal Care Committee of Qinghai University (No. QUA-2020-0710).

### 2.2 Experimental conditions and diet

The trial was conducted from April to July 2024 at XiangkaMeiduo Co., Ltd. (Qinghai, China). The experimental period lasted for 90 days. A total of 120 healthy male lambs (2 months old, initial body weight: 17.33 ± 0.21 kg) were randomly assigned to four dietary treatments (*n* = 30 per group) in a completely randomized design. The treatments received the same basal diet supplemented with 0% (CON), 0.08% (LG), 0.10% (MG), or 0.12% (HG) GAA. The GAA (content ≥ 98.0%, ash content ≤ 1.0%, moisture content ≤ 1.0%) was produced by Hebei Guangrui Animal Husbandry Co., Ltd. The composition and nutritional levels of the basal diet were presented in Table 1.

**Table 1:** Dietary concentrate composition and nutrient levels (dry matter basis).

Items	CON	LG	MG	HG
Ingredients (%)				
Corn	48.32	48.24	48.22	48.2
Wheat	9.00	9.00	9.00	9.00
Palm kernel meal	16.00	16.00	16.00	16.00
Soybean meal	4.00	4.00	4.00	4.00
Rapeseed meal	15.00	15.00	15.00	15.00
NaCl	1.00	1.00	1.00	1.00
CaCO3	1.00	1.00	1.00	1.00
NaHCO3	1.00	1.00	1.00	1.00
Betaine	0.08	0.08	0.08	0.08
Premix^a^	4.60	4.60	4.60	4.60
Guanidinoacetic acid	0.00	0.08	0.10	0.12
Total	100.00	100.00	100.00	100.00
**Analyzed composition**				
Dry matter (MJ/kg)^b^	12.16	12.15	12.15	12.15
Crude protein %^c^	14.68	14.67	14.67	14.67
Crude fiber %^c^	20.87	20.85	20.84	20.84
Ether extract %^c^	4.20	4.20	4.20	4.20
Neutral detergent fiber %^d^	20.89	20.89	20.88	20.88
Acid detergent fiber %^d^	13.92	13.92	13.91	13.91
Ca %^d^	0.88	0.88	0.88	0.88
P %^d^	0.44	0.44	0.44	0.44

^a^The premix provided per kilogram of diet: Cu (15 mg), Fe (55 mg), Zn (25 mg), Mn (40 mg), Se (0.30 mg), I (0.5 mg), Co (0.20 mg), vitamin A (20,000 IU), vitamin D (4,000 IU), and vitamin E (40 IU). ^b^Calculated according to the nutritional requirements for fattening sheep (NRC, 2007). ^c^These values were experimentally determined. ^d^These values were calculated according to the Chinese Feed Composition and Nutritive Value Table (31st edition, 2020).

The dietary roughage consisted of oat silage and oat hay (at a 1:1 ratio on a dry matter basis).

Tibetan sheep had *ad-libitum* access to feed and water, which fed twice daily at 8:00 am and 5:00 pm. At the end of the feeding trial, six lambs were randomly selected from each group and transported to a nearby commercial abattoir. In accordance with animal welfare protocols, all individuals were subjected to a 24 h fasting period and a 2 h water deprivation period prior to slaughter. The subcutaneous adipose was collected and stored at −80°C for the future analysis.

### 2.3 Measurement of subcutaneous fat thickness and histomorphological observation

Subcutaneous fat thickness was measured (*n* = 6 per group) at the 12th~13th rib intercostal space, 5 cm from the dorsal midline, and then adipose tissue samples were collected.

Subcutaneous adipose tissue samples were fixed in 4% formaldehyde solution for 24 h, dehydrated through a graded ethanol series (70%, 80%, 95%, 100%), embedded in paraffin, and sectioned at a thickness of 5 μm. The sections were stained with hematoxylin for 5 min, rinsed under running water for 10 min, then stained with 0.5% eosin for 3 min and rinsed with distilled water. Adipocyte morphology was systematically observed at 200 × magnification using a DP2-BSW digital microscope (Olympus, Tokyo, Japan).

### 2.4 Determination of adipose tissue antioxidant capacity

Antioxidant capacity was measured using enzyme-linked immunosorbent assay (ELISA) kits (Nanjing Jiancheng Bioengineering Institute, Nanjing, China) according to the manufacturer's instructions. The measured parameters included CAT, SOD, GSH-Px, MDA, and T-AOC. A standard curve was independently established for each microplate, and the correlation coefficient (R^2^) of the standard curve was required to be greater than 0.95.

### 2.5 Determination of fatty acid content

Accurately weigh 100 mg of adipose tissue sample and extract total lipids using the Folch method. Add 2 mL of 0.3 M potassium hydroxide in methanol to the lipid extract, saponify in a 95 °C water bath for 2 min, and then continue saponification at room temperature to fully cleave the fatty acyl chains. After saponification, add 2 mL of BF3-methanol solution and esterify in a 60 °C water bath for 30 min. After cooling, add 1 mL of hexane and 2 mL of NaCl solution, allow the mixture to stand for phase separation, and collect the upper hexane phase. Use the supernatant for analysis on a gas chromatograph (Agilent 7890B, Agilent Technologies, USA). Identify fatty acid methyl esters by comparing their retention times with those of standards. Calculate absolute contents using the internal standard method (C17:0).

### 2.6 RNA-seq analysis

Subcutaneous adipose tissue samples (*n* = 6 per group) were subjected to RNA-seq. Total RNA was extracted from Tibetan sheep adipose tissue using TRIzol reagent (Magen R4801-02). RNA integrity and concentration were assessed using an Agilent 2100 Bioanalyzer. mRNA with polyA tails was enriched with Oligo (dT) magnetic beads and randomly fragmented in the presence of divalent cations. First-strand cDNA was synthesized using the fragmented mRNA as template and random primers with M-Mu LV reverse transcriptase. The RNA strand was then degraded by RNase H, and second-strand cDNA was synthesized using DNA polymerase I. The purified double-stranded cDNA was end-repaired, A-tailed, and ligated with sequencing adapters. Fragments of 370–420 bp were selected and amplified by PCR. Paired-end 150 bp sequencing was performed on an Illumina NovaSeq 6000 platform (Illumina, San Diego, CA, USA).

Raw sequencing data were quality-checked with FastQC, and low-quality reads and adapter sequences were removed using Trimmomatic. Clean reads were aligned to the reference genome with HISAT2, and gene expression was quantified using featureCounts; Q20, Q30, and GC content were also calculated. Differentially expressed genes (DEGs) were identified using DESeq2 with thresholds of *P* < 0.05 and |log_2_ FC| > 1. WGCNA was conducted on the entire expression dataset consisting of all 24 samples (6 per group × 4 groups). Core module genes were screened based on phenotypic data, and KEGG pathway enrichment analysis was conducted for genes within the modules.

RT-qPCR validation was performed for the key differentially expressed genes. The qPCR reaction was carried out using SYBR Green Premix Ex Taq II (Takara) on a LightCycler 480 Real-Time PCR System (Roche, Basel, Switzerland). GAPDH served as the internal reference gene. Relative expression levels were calculated using the 2^−ΔΔ*Ct*^ method, with the control group as the calibrator. Detailed information on primer sequences, annealing temperatures, and PCR product lengths is provided in Table 2.

**Table 2:** Primers used in RT-qPCR.

Gene	Primer (5^′^to3^′^)	Product size (bp)	TM (°C)
*PIK3CB*	F: GATGCCCTTCTGAACTGGCT	92	60
	R: CAGTAGCCGGCACAGGATAG		
*ANGPT2*	F: AGCACGAAGGATGCAGACAA	101	60
	R: CCATTCAGGTTGGAGGGACC		
*PLA2G4A*	F: GCCATGGTAGGATTCTCCGG	90	60
	R: GGATCCGGAAAGACCAGCAA		
*IDNK*	F: AGCCAGTTGAGGTGAAGCTC	111	60
	R: ACTGCAGTAATTCAGGGGGC		
*MAPKAPK5*	F: CCCAAGAACGGTTTGCACTG	101	60
	R: AGCTCTCCCCCTTCCATCAT		
*GAPDH*	F: ACAGTCAAGGCAGAGAACGG	98	60
	R: CCAGCATCACCCCACTTGAT		

The melting temperature (Tm) of each primer pair is 60 °C, ensuring optimal annealing during PCR.

### 2.7 LC-MS/MS analysis

This study used LC-MS/MS to investigate the effects of GAA on lipid metabolites in subcutaneous adipose tissue (*n* = 6 per group). 100 mg of adipose tissue was mixed with 10 mL of pre-chilled methyl tert-butyl ether (MTBE)/methanol (3:1, v/v) containing 10 μL of SPLASH LIPIDOMIX (Avanti Polar Lipids, USA) as an internal standard. The sample was homogenized and then shaken at 4 °C for 30 min. Ultrapure water was added to induce phase separation, followed by centrifugation at 12,000 × g and 4 °C for 10 min. The upper organic phase was collected and evaporated under nitrogen. The residue was reconstituted in isopropanol/methanol (1:1, v/v), filtered through a 0.22 μm membrane, and transferred to an autosampler vial. To assess method stability, equal aliquots of all sample supernatants were pooled to prepare quality control (QC) samples, which were inserted every six analytical runs.

Chromatographic separation was performed on a UHPLC system (Thermo Fisher Scientific, Waltham, MA, USA) equipped with an ACQUITY BEH C18 column (100 × 2.1 mm, 1.7 μm, Waters, Milford, MA, USA). The column temperature was maintained at 55 °C, and the flow rate was 0.3 mL/min. Mobile phase A consisted of acetonitrile/water (60:40, v/v) containing 10 mM ammonium formate and 0.1% formic acid, while mobile phase B consisted of isopropanol/acetonitrile (90:10, v/v) containing 10 mM ammonium formate and 0.1% formic acid. The injection volume was 5 μL.

Mass spectrometry was carried out using a Q Exactive HF-X high-resolution mass spectrometer (Thermo Fisher Scientific, Waltham, MA, USA) equipped with an electrospray ionization (ESI) source operating in both positive and negative ion modes. The spray voltage was 3.5 kV in positive mode and 2.8 kV in negative mode. The ion transfer tube temperature was 320 °C, and the auxiliary gas heater temperature was 350 °C. Data-dependent acquisition (DDA) was used to obtain MS/MS spectra. Full-scan MS was acquired over the m/z range 200~2,000 at a resolution of 120,000, and MS/MS scans were acquired at a resolution of 30,000. The top five most intense precursor ions were selected for fragmentation using stepped normalized collision energies of 20, 30, and 40 eV.

Raw LC-MS data were processed using Progenesis QI software (version 2.4, Non-linear Dynamics, Newcastle, UK). Lipid metabolites were identified by matching against public databases (LipidBlast, LIPID MAPS, MassBank) and an in-house standard database. Data were normalized by total peak area before statistical analysis. Principal component analysis (PCA) and partial least-squares discriminant analysis (PLS-DA) were performed to evaluate sample grouping and intergroup differences. Differential lipids were selected based on VIP > 1 and *P* < 0.05. Functional enrichment of differential lipids was determined using KEGG pathways, with *P* < 0.05 considered significantly enriched.

### 2.8 Statistical analysis

Data on antioxidant capacity and fatty acid content were analyzed using SPSS 26.0. One-way analysis of variance (ANOVA) was first performed to compare overall differences among the four treatment groups, followed by polynomial contrasts to partition the between-group variation into linear and quadratic trend components, thereby revealing the dose–response relationships of these parameters with increasing addition levels. All tests adopted *P* < 0.05 as the criterion for statistical significance. Pearson correlation analysis was conducted using Origin 2021 software (OriginLab Corporation, Northampton, MA, USA).

## 3 Results

### 3.1 Effects of GAA on Subcutaneous Fat Thickness and Tissue Morphology

**Figure 1:**
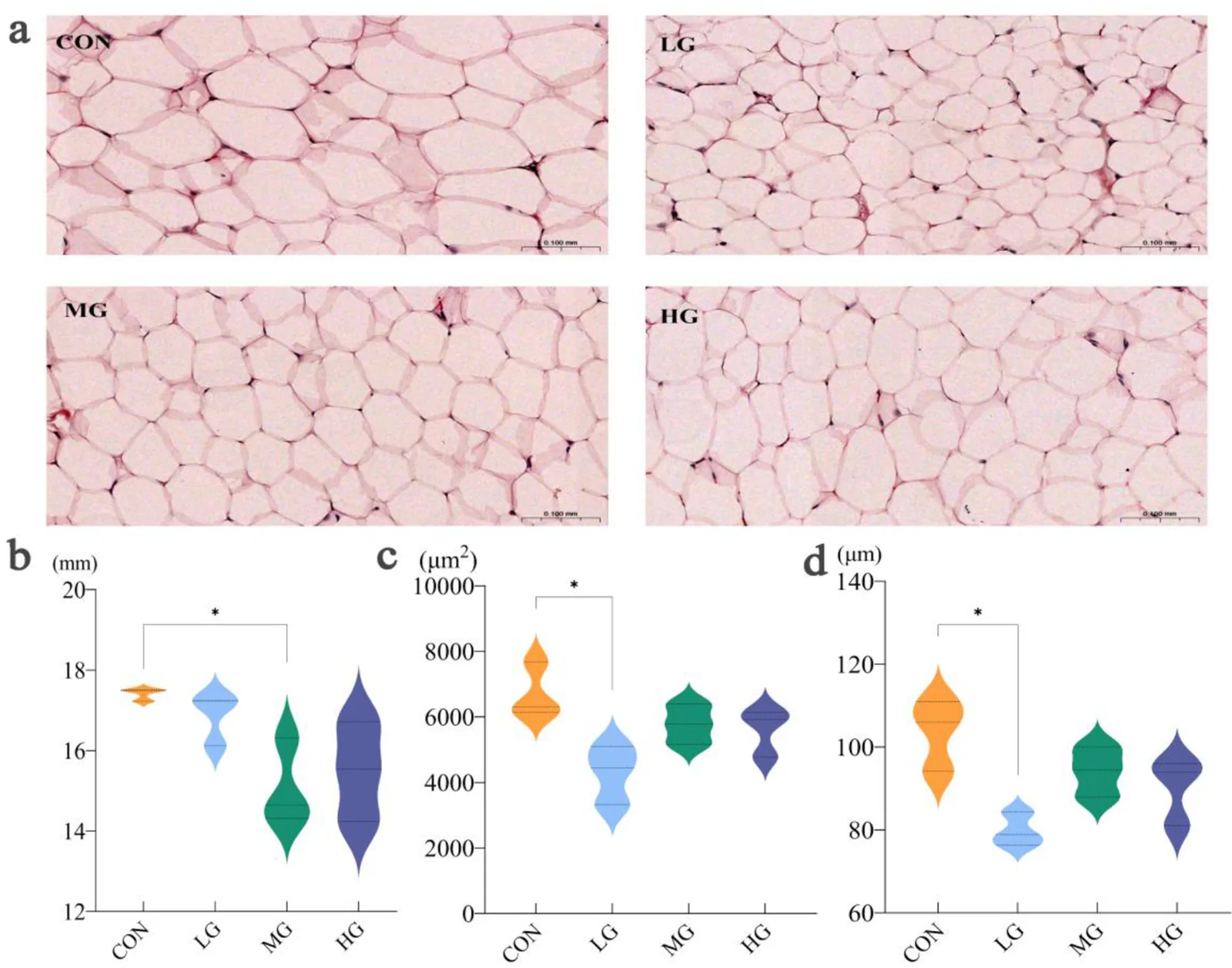
Effects of GAA on subcutaneous fat thickness and tissue morphology in Tibetan sheep. **(a)** H&E-stained sections of adipose tissue from the four groups under 100 μm field of view. **(b–d)** Measurements of subcutaneous fat thickness, adipocyte area, and adipocyte diameter across the four groups.

Observation of H&E-stained sections displayed the phenotype of subcutaneous adipose tissue (Figure 1a). Compared to the CON group, the lambs in the MG group exhibited a reduction in subcutaneous fat thickness (*P* < 0.05) (Figure 1b). Additionally, both adipocyte area and adipocyte diameter were significantly reduced in the LG group compared to the CON (*P* < 0.05) (Figures 1c, d).

### 3.2 Effects of GAA on the antioxidant capacity of subcutaneous adipose tissue

As shown in Table 3, the MDA content was lowest in the MG group, significantly lower than that in the CON, LG, and HG groups (*P* < 0.05), and exhibited a quadratic trend (an initial decrease followed by an increase) with treatment level (*P-*quadratic < 0.05). The highest SOD activity was observed in the MG group, significantly exceeding that in all other groups (*P* < 0.05). Similarly, T-AOC peaked in the MG group, with levels significantly higher than those in the CON and LG groups (*P* < 0.05). Both variables exhibited significant linear and quadratic trends (*P*-linear, *P*-quadratic < 0.05). In conclusion, the 0.10% GAA treatment effectively reduced the MDA content, increased SOD activity and T-AOC level, and exhibited the optimal antioxidant protective effect.

**Table 3:** Antioxidant index assays.

Items	Groups				SEM	***P***		
	**CON**	**LG**	**MG**	**HG**		**ANOVA**	**Linear**	**Quadratic**
CAT (U/mg)	23.67	23.09	24.57	23.70	0.22	0.097	0.353	0.698
GSH-Px (U/mg)	134.61	129.90	137.25	131.31	2.12	0.674	0.903	0.895
MDA (nmol/mg)	4.93^a^	4.31^a^	2.55^b^	3.96^a^	0.30	0.006	0.014	0.016
SOD (U/mg)	59.10^b^	58.75^b^	73.37^a^	63.29^b^	1.92	0.001	0.008	0.022
T-AOC (mmol/g)	6.86^c^	8.36^b^	9.66^a^	9.03^ab^	0.33	< 0.001	< 0.001	0.004

a, b Within the same row, means bearing different superscript letters differ significantly (*P* < 0.05). CAT, catalase; SOD, superoxide dismutase; MDA, malondialdehyde; GSH-Px, glutathione peroxidase; T-AOC, total antioxidant capacity.

### 3.3 Effects of GAA on the composition and content of fatty acids in subcutaneous adipose tissue

The fatty acid profile of subcutaneous fat was analyzed, and a total of 40 fatty acids were detected, including 14 saturated fatty acids (Table 4) and 26 unsaturated fatty acids (Table 5). Among the saturated fatty acids, the C16:0 content was highest in the LG group, significantly higher than that in the CON, MG, and HG groups (*P* < 0.05). The contents of C11:0, C13:0, and C15:0 did not differ significantly among groups (*P* > 0.05), but all exhibited significant quadratic trends (*P-*quadratic < 0.05), characterized by an initial increase followed by a decrease with increasing treatment level.

**Table 4:** Composition and content of saturated fatty acids (ng/g).

**Items**	Groups				SEM	***P***		
	**CON**	**LG**	**MG**	**HG**		**ANOVA**	**Linear**	**Quadratic**
C8:0	100.13	98.43	102.20	74.39	6.05	0.359	0.199	0.297
C10:0	745.41	830.26	964.11	570.01	68.89	0.241	0.506	0.094
C11:0	43.12	63.40	61.98	38.00	4.71	0.106	0.639	0.021
C12:0	2,240.64	2,304.53	1,868.92	2,607.76	135.23	0.314	0.578	0.226
C13:0	79.83	118.89	104.86	80.18	6.87	0.090	0.797	0.020
C14:0	7,369.15	9,050.10	7,254.10	8,105.41	563.04	0.715	0.922	0.756
C15:0	1,458.80	2,038.67	1,851.47	1,539.62	100.27	0.122	0.943	0.028
C16:0	40,593.27^b^	58,962.82^a^	39,182.46^b^	45,300.18^b^	3,116.66	0.038	0.798	0.236
C17:0	2,264.18	3,377.41	2,993.12	2,788.26	192.34	0.234	0.470	0.097
C18:0	37,481.5	62,499.13	43,895.12	54,962.38	4,939.75	0.305	0.442	0.477
C20:0	734.43	1,136.94	714.01	1,130.58	143.99	0.632	0.590	0.982
C22:0	215.60	272.43	168.93	236.03	36.99	0.843	0.912	0.952
C23:0	256.75	309.74	170.52	297.84	45.71	0.759	0.972	0.720
C24:0	644.52	739.58	506.17	683.64	89.78	0.864	0.901	0.843

a, b Within the same row, means bearing different superscript letters differ significantly (*P* < 0.05). HG: basal diet supplemented with 0.12% GAA, MG: basal diet supplemented with 0.10% GAA, LG: basal diet supplemented with 0.08% GAA, CON, basal diet.

**Table 5:** Composition and content of unsaturated fatty acids (ng/g).

**Items**	Groups				SEM	***P***		
	**CON**	**LG**	**MG**	**HG**		**ANOVA**	**Linear**	**Quadratic**
C14:1	826.17	961.36	840.34	990.45	73.76	0.856	0.628	0.965
C15:1(n-5)	24.12	21.69	36.55	22.45	3.96	0.571	0.797	0.499
C15:1(n-5) T	52.32	78.58	69.50	60.37	8.27	0.761	0.857	0.357
C16:1(n-7)	4,846.49	5,198.69	3,888.98	4,441.07	454.90	0.817	0.591	0.924
C18:1(n-12) T	28,296.29	35,783.49	31,435.99	28,628.96	1,416.70	0.219	0.777	0.079
C18:1(n-7)	4,811.25	5,488.36	4,832.06	4,397.96	355.79	0.806	0.605	0.500
C18:1(n-9)	4,894.75	6,621.88	5,682.54	5,064.69	428.12	0.536	0.916	0.221
C18:1(n-9) T	30,034.88	37,317.65	31,189.79	30,564.64	1,436.49	0.256	0.712	0.174
C18:2(n-6)	10,662.11^ab^	11,231.27^a^	10,032.20^ab^	9,085.85^c^	321.71	0.032	0.030	0.170
C18:3(n-6)	875.04	948.87	804.73	790.97	41.10	0.566	0.330	0.622
C19:1(n-12) T	228.90	313.53	300.12	286.92	29.88	0.813	0.602	0.481
C18:3(n-3)	2,284.82^ab^	2,937.78^a^	2,398.77^ab^	1,916.35^c^	150.97	0.041	0.084	0.047
C20:1(n-9)	350.47	488.79	328.64	413.35	43.53	0.627	0.947	0.778
C20:1(n-9) T	1,512.87	1,622.51	1,386.95	1,503.61	104.68	0.918	0.811	0.989
C20:3(n-6)	841.91	896.98	996.77	689.73	67.20	0.491	0.575	0.222
C20:2(n-6)	411.00	452.82	473.45	507.63	33.38	0.827	0.377	0.960
C20:3(n-3)	969.87	880.08	962.65	625.44	65.69	0.215	0.110	0.326
C20:4(n-6)	17,232.13	19,123.70	17,617.69	13,563.34	1,849.16	0.802	0.513	0.488
C20:5(n-3)	3,361.22	3,397.53	4,048.23	3,379.01	330.41	0.893	0.838	0.649
C22:1(n-9)	168.21	214.35	109.61	146.16	31.19	0.748	0.590	0.946
C22:1(n-9) T	701.33	696.21	375.36	726.54	86.62	0.481	0.763	0.340
C22:2(n-6)	31.32^a^	31.38^a^	20.39^b^	26.11^ab^	1.70	0.033	0.037	0.269
C22:5(n-3)	420.96	693.27	696.13	391.35	85.66	0.468	0.914	0.134
C22:5(n-6)	4,586.65	4,542.98	5,005.33	3,458.17	386.14	0.597	0.443	0.381
C22:6(n-3)	1,110.94	1,630.10	1,826.78	899.95	175.85	0.210	0.765	0.051
C24:1(n-9)	412.45	410.60	243.75	398.39	40.51	0.429	0.578	0.360
n-6 PUFA	34,640.16	37,228.01	34,950.56	28,121.80	2,319.92	0.609	0.347	0.363
n-3 PUFA	8,147.81	9,538.76	9,932.55	7,212.09	546.41	0.285	0.613	0.080
n-6/n-3	4.25	3.84	3.59	3.93	0.16	0.592	0.440	0.290

a, b Within the same row, means bearing different superscript letters differ significantly (*P* < 0.05). HG: basal diet supplemented with 0.12% GAA, MG: basal diet supplemented with 0.10% GAA, LG: basal diet supplemented with 0.08% GAA, CON, basal diet. T, T denotes the trans configuration of fatty acids.

Among the unsaturated fatty acids, the C18:2(n-6) content was significantly lower in the HG group than in the MG, LG, and CON groups (*P* < 0.05), and it showed a significant linear decreasing trend with increasing treatment level (*P-*linear < 0.05). The C18:3(n-3) content was also significantly lower in the HG group than in the MG, LG, and CON groups (*P* < 0.05), and it exhibited a significant quadratic trend, increasing initially and then decreasing (*P-*quadratic < 0.05). The C22:2(n-6) content was significantly lower in the MG group than in the LG and CON groups (*P* < 0.05), with a significant linear decreasing trend (*P-*linear < 0.05).

### 3.4 Effects of GAA on the gene expression profile of subcutaneous adipose tissue

**Figure 2:**
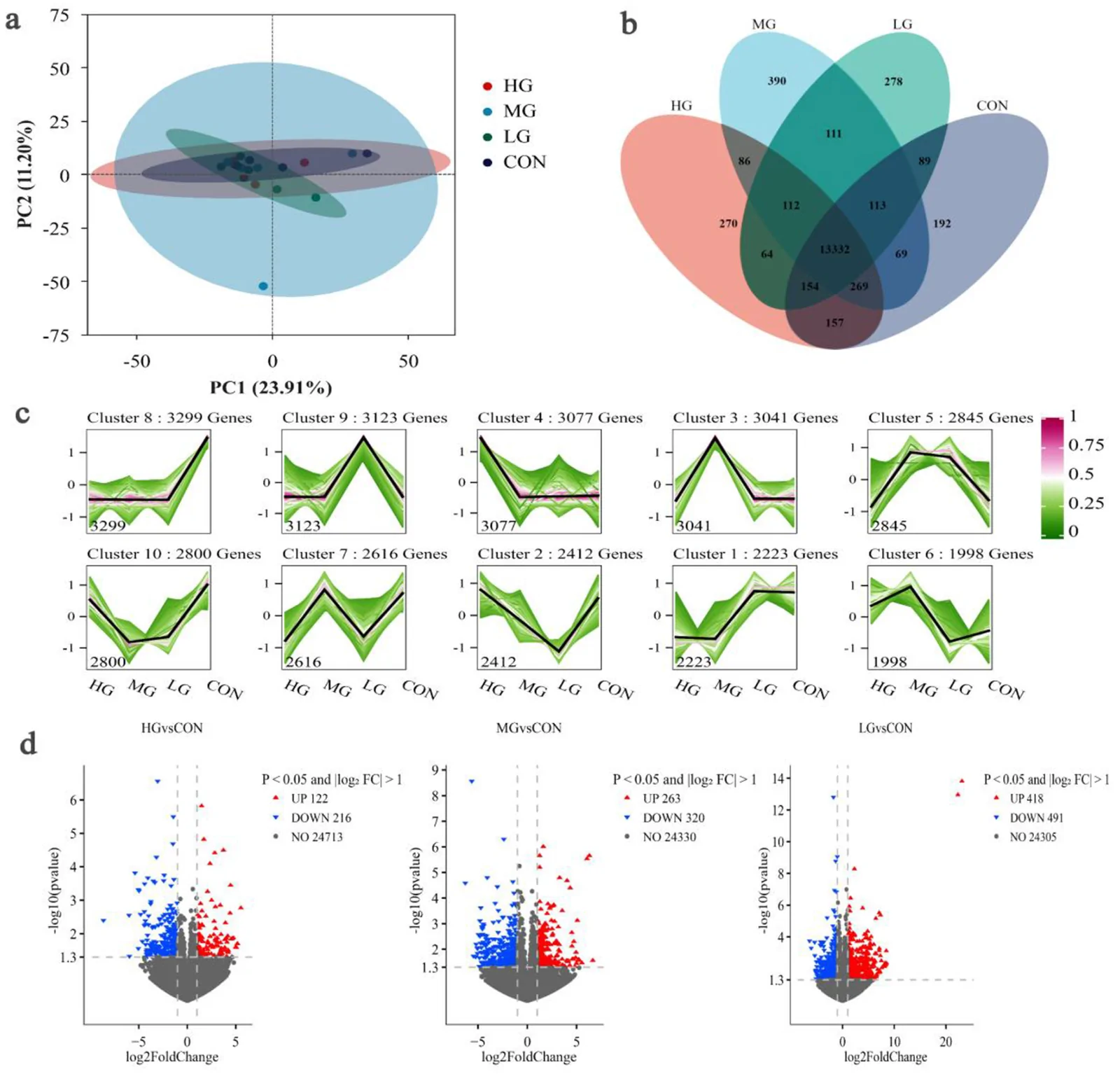
Gene Expression Characteristics of RNA-seq Results. **(a)** PCA plot showing the separation of RNA-seq profiles among the four groups. **(b)** Venn diagram illustrating the overlap of genes across the four groups. **(c)** Trend plot displaying gene expression patterns divided into 10 distinct profiles. **(d)** Volcano plots display the distribution of differentially expressed genes (DEGs) in the HG vs. CON, MG vs. CON, and LG vs. CON comparisons, respectively. Red represents significantly upregulated genes, while blue represents significantly downregulated genes.

Raw sequencing generated 211.9 Gb of data, of which 164.79 Gb were retained after quality filtering. For all samples, Q20 scores exceeded 97.67%, Q30 scores exceeded 93.49%, and the average GC content was 50.19% (Supplementary Table S1). PCA of all expressed genes showed clear separation among the groups (Figure 2a). A total of 14,444, 14,482, 14,253, and 14,375 genes were identified in the HG, MG, LG, and CON groups, respectively, with 13,332 genes common to all four groups (Figure 2b). Trend analysis revealed 10 gene sets with distinct expression patterns (Figure 2c). Applying the thresholds *P* < 0.05 and |log_2_ FC| > 1, differential expression analysis identified 338 (122 upregulated, 216 downregulated), 583 (263 upregulated, 320 downregulated), and 909 (418 upregulated, 491 downregulated) DEGs in the HG vs. CON, MG vs. CON, and LG vs. CON comparisons, respectively (Figure 2d).

**Figure 3:**
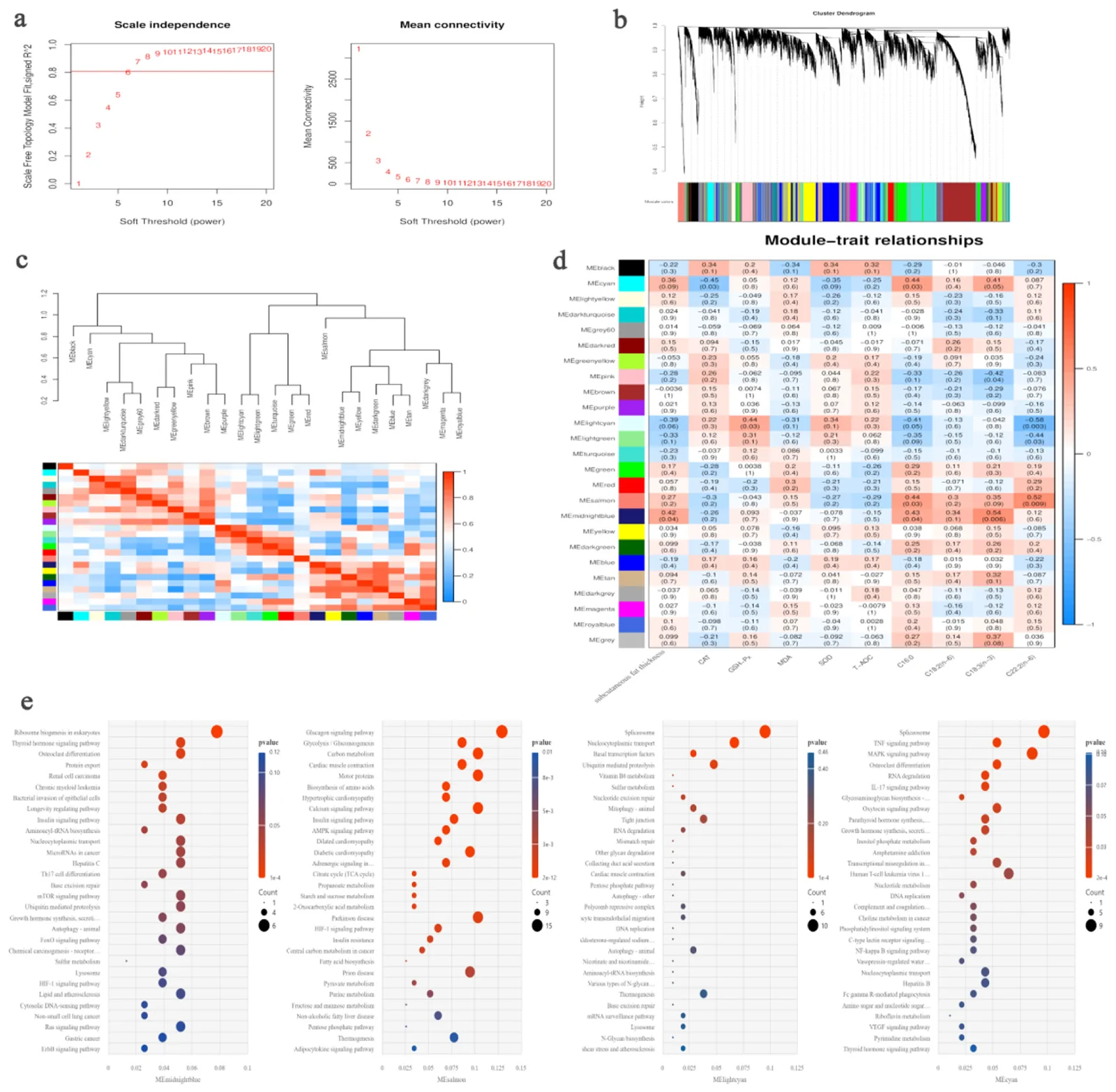
WGCNA and KEGG pathway enrichment analysis. **(a)** Selection of the soft-thresholding power, showing the relationship between power values and scale-free topology fit **(left)** and mean connectivity **(right)**. **(b)** Hierarchical clustering dendrogram of genes. **(c)** Cluster dendrogram showing the correlation between identified modules. **(d)** Module-trait relationship heatmap, displaying correlations between module eigengenes and phenotypic traits. **(e)** KEGG pathway enrichment analysis and the top 30 enriched pathways for all genes in the MEmidnightblue, MEsalmon, MElightcyan, and MEcyan modules.

To investigate the effect of GAA on gene expression in subcutaneous adipose tissue and to identify key genes associated with subcutaneous fat thickness, antioxidant enzyme activities, and fatty acid profiles, we performed WGCNA. Based on the scale-free topology criterion, a soft-thresholding power of 6 was selected (R^2^ > 0.8; mean connectivity moderate), indicating that the network satisfied the requirements for module identification (Figure 3a). Using the dynamic tree cut method, we identified 25 co-expression modules (Figure 3b) and then analyzed correlations among them (Figure 3c). Module–trait correlation analysis (Figure 3d) revealed the following: the MEmidnightblue module was significantly positively correlated with subcutaneous fat thickness, C16:0, and C18:3(n-3). The MEsalmon module was significantly positively correlated with C22:2(n-6) and C16:0. The MEcyan module was positively correlated with C16:0 and C18:3(n-3) but negatively correlated with the activities of CAT and SOD. And the MElightcyan module was positively correlated with GSH-Px activity and negatively correlated with C22:2(n-6) and C16:0. Genes belonging to these four modules were subjected to KEGG pathway enrichment analysis, and the top 30 enriched pathways were displayed (Figure 3e). Several pathways including the insulin signaling pathway, mTOR signaling pathway, pentose phosphate pathway (PPP), and MAPK signaling pathway were significantly enriched and were highly relevant to the phenotypic traits observed in this study.

### 3.5 Effects of GAA on the lipid metabolite profile

**Figure 4:**
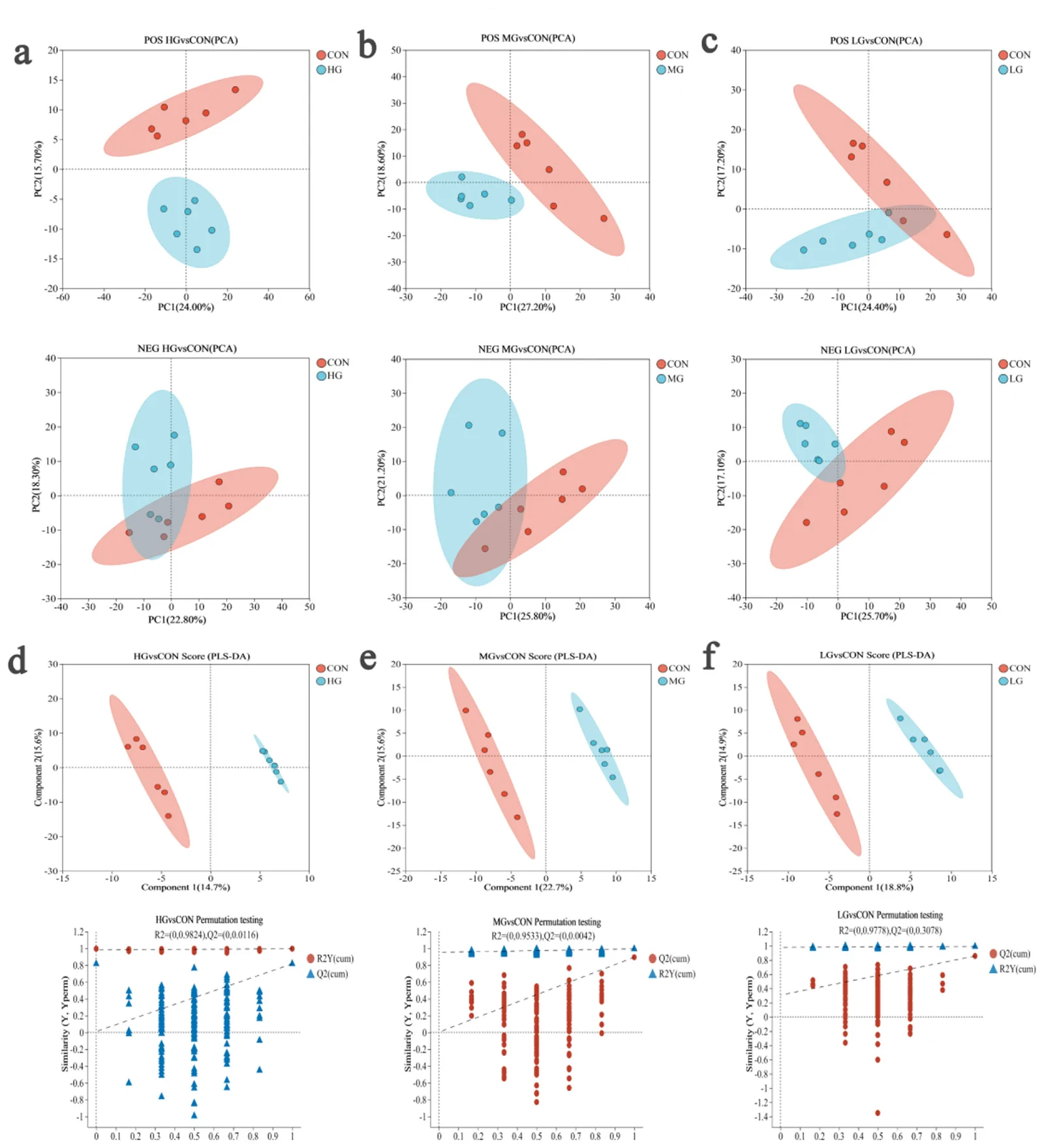
Model Quality Validation Based on LC-MS/MS Data. **(a–c)** PCA score plots for the HG vs. CON, MG vs. CON, and LG vs. CON comparisons in positive and negative ion modes, respectively. **(d–f)** PLS-DA score plots and their corresponding permutation test validation for the HG vs. CON, MG vs. CON, and LG vs. CON comparisons.

After lipid metabolites with RSD > 30% in pooled QC samples were removed, 606 and 550 metabolites were identified in the positive and negative ion modes, respectively. The RSDs of the QC samples were shown in Supplementary Table S2, and the median CVs for each lipid class were listed in Supplementary Table S3. PCA of these metabolites revealed a clear separation among the three comparison groups in both the positive (Figures 4a, b) and negative (Figure 4c) ion modes. PLS-DA was then performed on the combined dataset from both ionization modes to model the relationship between lipid metabolite expression and sample groups. Model validation was conducted using a 200-iteration permutation test; the resulting Q^2^ and R^2^ values indicated no overfitting, confirming that the model was reliable for sample characterization (Figures 4d–f).

**Figure 5:**
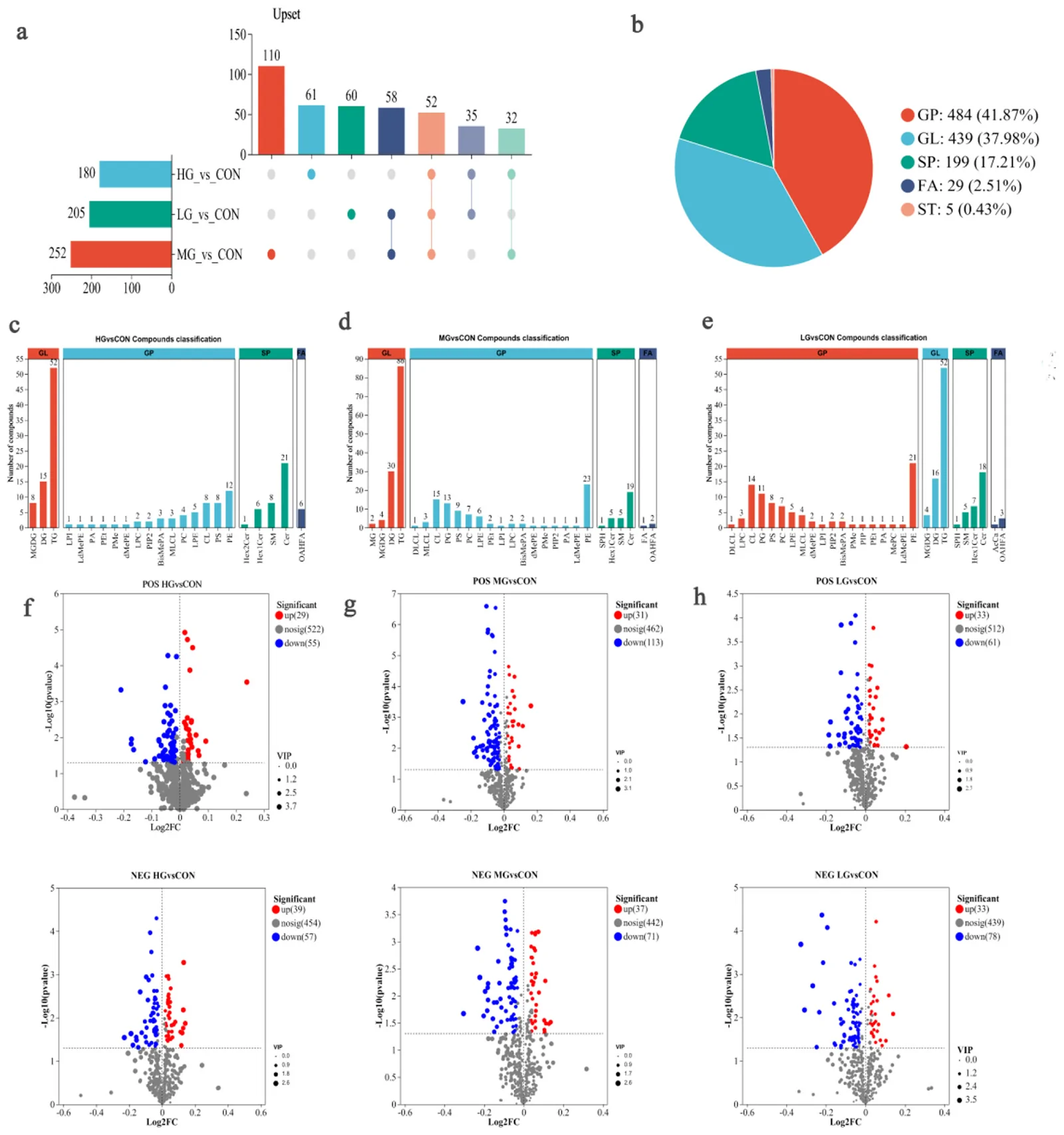
Analysis of differential lipid metabolites. **(a)** Upset plot illustrating the number of lipid metabolites across the three comparison groups. **(b)** Pie chart showing the classification and annotation results of lipid metabolites from the three comparison groups. **(c–e)** Bar charts displaying the classification and annotation results of lipid metabolites for the HG vs. CON, MG vs. CON, and LG vs. CON comparisons, respectively. **(f–h)** Volcano plots showing the distribution of differential metabolites for the HG vs. CON, MG vs. CON, and LG vs. CON comparisons, respectively. Red represents significantly upregulated metabolites, while blue represents significantly downregulated metabolites.

An UpSet plot illustrated the intersection of lipid molecules across the comparison groups, revealing 61, 110, and 60 unique lipids in the HG vs. CON, MG vs. CON, and LG vs. CON comparisons, respectively, with 52 lipids shared among all three groups (Figure 5a). Lipid annotation identified five major classes: Glycerophospholipids (41.87%), Glycerolipids (37.98%), Sphingolipids (17.21%), Fatty acids (2.51%), and Sterol Lipids (0.43%) (Figure 5b). Glycerolipids predominated in both the HG vs. CON and MG vs. CON comparisons, with triglycerides as the most abundant subclass (52 and 86 molecules, respectively) (Figures 5c, d). In the LG vs. CON comparison, glycerophospholipids constituted the largest class, with 98 metabolites detected (Figure 5e). Volcano plots were used to visualize the overall distribution of differential lipid metabolites. Based on the thresholds of *P* < 0.05 and VIP > 1, the differential metabolites identified in the positive and negative ion modes, respectively, were as follows: 84 (29 upregulated, 55 downregulated) and 96 (39 upregulated, 57 downregulated) in the HG vs. CON comparison (Figure 5f); 114 (31 upregulated, 83 downregulated) and 108 (37 upregulated, 71 downregulated) in the MG vs. CON comparison (Figure 5g); and 94 (33 upregulated, 61 downregulated) and 111 (33 upregulated, 78 downregulated) in the LG vs. CON comparison (Figure 5h).

**Figure 6:**
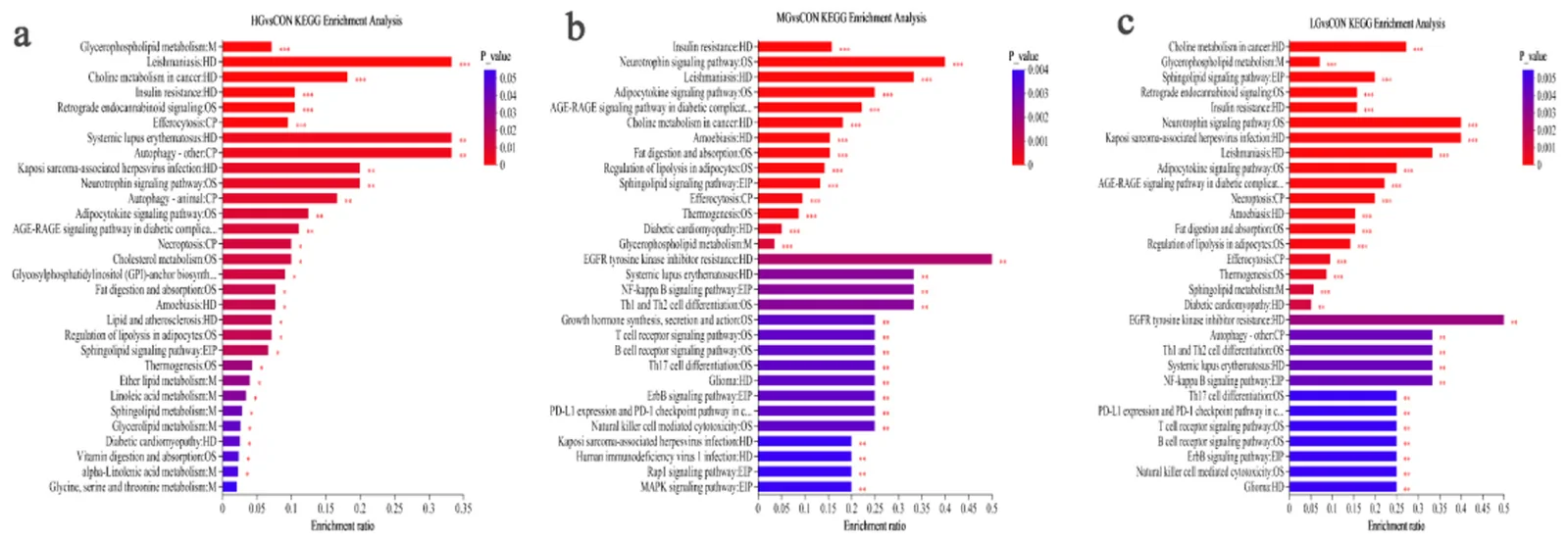
KEGG pathway enrichment analysis of differential lipid metabolites. **(a–c)** KEGG pathway enrichment analysis of differential metabolites for the HG vs. CON, MG vs. CON, and LG vs. CON comparisons, respectively, displaying the top 30 enriched pathways.

Differential lipid metabolites were subjected to KEGG pathway enrichment analysis, and the top 30 significantly enriched pathways (*P* < 0.05) were displayed for each comparison (Figure 6). In the HG vs. CON group, significant enrichment was observed in pathways including alpha linolenic acid metabolism, linoleic acid metabolism, the adipocytokine signaling pathway, regulation of lipolysis in adipocytes, fat digestion and absorption, and glycerophospholipid metabolism (Figure 6a). In the MG vs. CON group, enriched pathways comprised fat digestion and absorption, the adipocytokine signaling pathway, and glycerophospholipid metabolism (Figure 6b). In the LG vs. CON group, significant enrichment was found in the regulation of lipolysis in adipocytes, fat digestion and absorption, glycerophospholipid metabolism, and the adipocytokine signaling pathway (Figure 6c).

### 3.6 Correlation analysis

**Figure 7:**
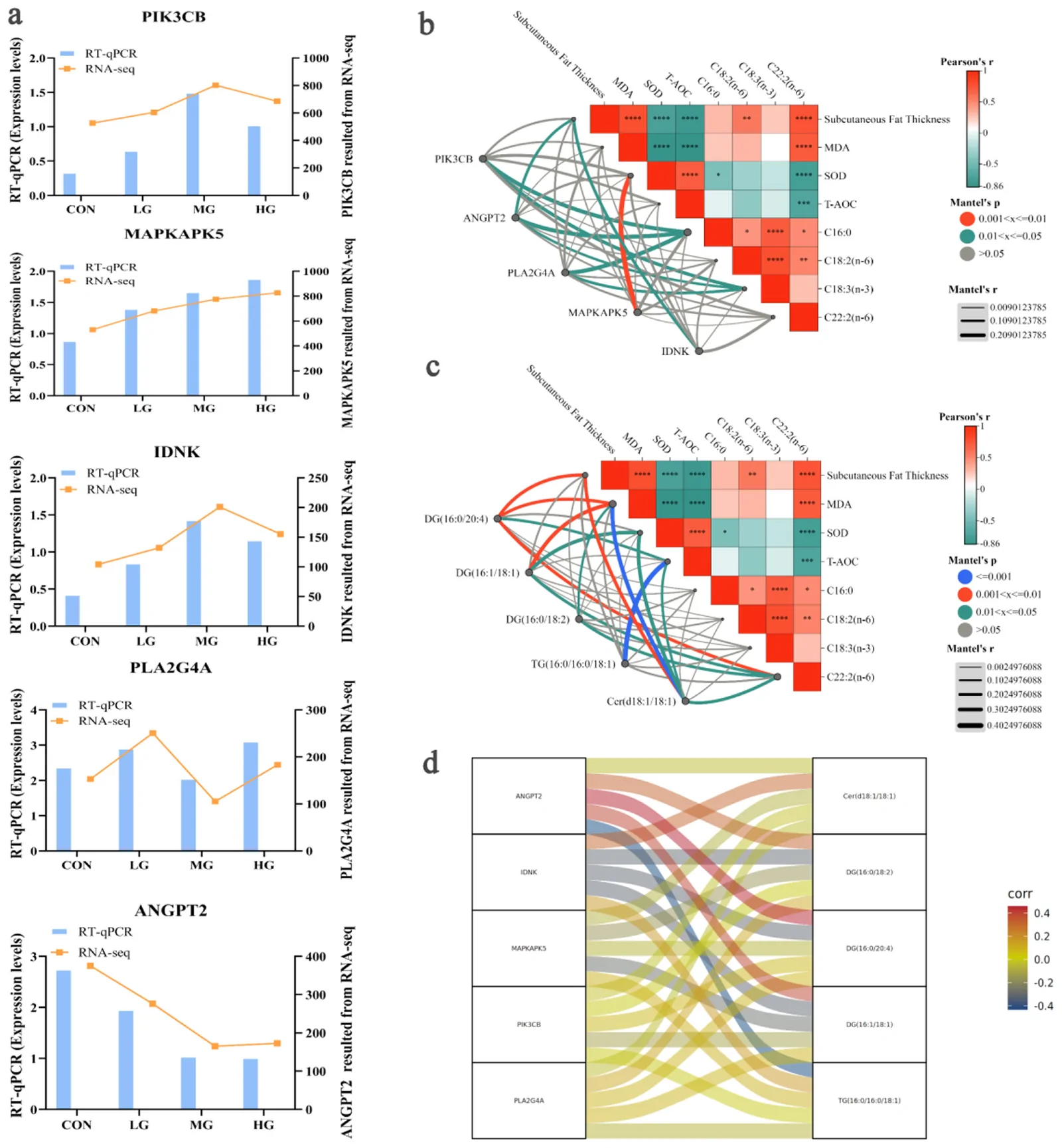
Correlation analysis and results of RT-qPCR. **(a)** RT-qPCR results of key differentially expressed genes. **(b)** Correlation analysis among key differentially expressed genes and phenotypic traits. **(c)** Correlation analysis among key differential metabolites and phenotypic traits. **(d)** Correlation analysis among key differential metabolites and key differential metabolites.

The regulation of subcutaneous fat metabolism involved multiple genes and lipid molecules. Pearson correlation analysis was performed among the differentially expressed genes (DEGs), phenotypes, and differential lipid metabolites (DAMs) to investigate their interrelationships. The DEGs including *PIK3CB, MAPKAPK5, IDNK, ANGPT2*, and *PLA2G4A*, along with the DAMs including DG (16:0/20:4), DG (16:1/18:1), DG (16:0/18:2), TG (16:0/16:0/18:1), and Cer (d18:1/18:1), were closely associated with the antioxidant capacity and metabolism of subcutaneous fat. RT-qPCR validation of the DEGs produced results consistent with the RNA-seq data (Figure 7a).

In the DEG-phenotype correlation analysis, *MAPKAPK5* was strongly positively correlated with SOD activity, *ANGPT2* was moderately positively correlated with subcutaneous fat thickness, and *PIK3CB* was moderately positively correlated with C18:3(n-3) content (Figure 7b).

In the DAM-phenotype correlation analysis, DG (16:0/20:4) and Cer (d18:1/18:1) were significantly positively correlated with MDA content, whereas TG (16:0/16:0/18:1) was significantly positively correlated with T-AOC level (Figure 7c).

In the DEG-DAM correlation analysis, *ANGPT2* was strongly positively correlated with DG (16:0/20:4) and DG (16:1/18:1), and strongly negatively correlated with TG (16:0/16:0/18:1). *IDNK* was strongly positively correlated with Cer (d18:1/18:1) (Figure 7d).

## 4 Discussion

Subcutaneous fat is one of the primary forms of adipose storage in animals, serving as a key fat depot that contributes to immune defense and mechanical protection (11, 12). However, excessive deposition of subcutaneous fat disadvantaged the feed conversion ratio and carcass characteristics, as well as changed in meat quality (13). Previous study found that the supplementation of GAA significantly reduced the porcine back fat thickness through regulating expression of lipid metabolism-related genes (*CPT-1* and *FAS*) (14). Broiler chickens fed with GAA showed a reduction in fat content of breast meat (15). Consistent with these findings, it was demonstrated that the dietary 0.10% GAA supplementation decreased deposition of subcutaneous fat in Tibetan sheep. Furthermore, supplementation with 0.10% GAA in diet increased the expression of uncoupling protein 3 (*UCP3*), whereas decreased the expression of stearoyl-coenzyme A desaturase (*SCD*) in subcutaneous fat. The development of obesity was promoted when knockout the *UCP3* in rat, which associated with upregulation of genes encoding enzymes from the transsulfuration pathway (16). *SCD* converted the stearoyl-coenzyme A to oleoyl-coenzyme A, which was involved the lipogenesis and potentiation of adiponectin signaling via modulating the peroxisomal proliferator-activated receptor γ (PPARγ) (17). Therefore, we proposed that dietary 0.10% GAA supplementation altered the lipid-related genes (*UCP3* and *SCD*), reducing subcutaneous fat in Tibetan sheep. Final body weight (FBW), average daily gain (ADG), and feed conversion ratio (FCR) are important factors that influence subcutaneous fat thickness. As detailed in the response letter, these data could not be included in this study.

Antioxidant capacity is normally maintained in a balanced state in mammals (18). In the present study, dietary supplementation with GAA increased SOD activity and T-AOC while decreasing MDA content, with the most pronounced effects observed at a supplementation level of 0.10%. This finding is consistent with a previous report that GAA enhanced the activities of T-AOC, SOD, CAT, and GSH-Px and reduced MDA concentration in the rumen of lambs (19). The improvement in antioxidant capacity may be associated with the upregulation of *IDNK* expression: *IDNK* functions in the PPP, a principal source of NADPH. NADPH supplies the reducing equivalents required to maintain glutathione and thioredoxin in their reduced states, thereby preserving intracellular redox homeostasis (20). The decreased MDA content indicated effective suppression of lipid peroxidation (21). Furthermore, MDA content exhibited a strong positive correlation with the level of DG (16:0/20:4). Because DG (16:0/20:4) is rich in arachidonic acid, which readily undergoes lipid peroxidation to yield MDA under oxidative stress (22). We propose that GAA and its metabolites inhibit this peroxidation process, thereby reducing MDA accumulation (23).

Moderate intake of unsaturated fatty acids confers multiple health benefits (24, 25), whereas excessive consumption of saturated fatty acids is an established risk factor for cardiovascular disease (26). In the present study, supplementation with 0.10% GAA significantly elevated the levels of C18:2(n-6) and C18:3(n-3) while significantly reducing the level of C16:0. Elevated C16:0 is associated with higher blood low-density lipoprotein (LDL) cholesterol and increased cardiovascular risk (27). In contrast, C18:2(n-6) lowers both total and LDL cholesterol, thereby conferring cardiovascular protection (28). C18:3(n-3) can be endogenously converted into eicosapentaenoic acid (EPA) and docosahexaenoic acid (DHA); consistent with this, EPA and DHA levels were highest in the 0.10% GAA group. EPA possesses anti-inflammatory, antithrombotic, and vasodilatory properties (29). DHA is essential for fetal neurodevelopment, infant and childhood cognitive maturation, and the maintenance of cognitive function in adults (30). Furthermore, we found that GAA supplementation upregulated the expression of *SLC27A6*. Because *SLC27A6* functions primarily to enhance cellular uptake of long-chain fatty acids (31), its upregulation may account for the significant increases in C18:2(n-6) and C18:3(n-3).

RNA-seq analysis of subcutaneous adipose tissue revealed that GAA supplementation upregulated *PIK3CB*, a gene involved in the insulin signaling pathway. Although upregulation of *PIK3CB* indicates enhanced insulin signaling and lipogenesis, subcutaneous fat thickness decreased in the experimental group. This finding suggests that improved insulin sensitivity and energy homeostasis accelerated systemic lipid turnover and redistribution, preferentially partitioning lipids into energy expenditure or intramuscular deposition rather than being subcutaneous storage. A positive correlation between *PIK3CB* and C18:3(n-3) content indicated a potential functional link between the insulin signaling and polyunsaturated fatty acids metabolism. *PIK3CB* upregulation reflects efficient insulin signal transduction (32), and normal insulin sensitivity is critical for maintaining lipid metabolic homeostasis (33). Under these conditions, C18:3(n-3) is less susceptible to oxidative consumption and accumulates more stably in adipose tissue. Since mammals cannot synthesize n-3 PUFAs and the basal diet lacks these fatty acids, the elevated C18:3(n-3) content is attributable to GAA-enhanced bile secretion, which enables to bypass biohydrogenation (34).

*MAPKAPK5* is a downstream effector kinase in the MAPK signaling pathway (35). Its upregulation indicates MAPK pathway activation and triggers adipocyte differentiation and adaptive remodeling, resulting in more complete differentiation and enhanced metabolic capacity in mature adipocytes (36). The methylation of GAA to creatine consumes S-adenosyl methionine (SAM), depleting the primary methyl donor pool (37). This depletion disrupts methylation homeostasis and indirectly activates the MAPK signaling pathway, promoting the differentiation of preadipocytes into functionally mature adipocytes (38). *MAPKAPK5* expression showed a strong positive correlation with SOD content. MAPK pathway promotes *SOD2* transcription (39). *SOD2* is a mitochondrial matrix enzyme that scavenges superoxide anions generated by the mitochondrial respiratory chain (40), thereby protecting adipocytes from oxidative damage during periods of enhanced anabolic activity and mitochondrial oxidative phosphorylation (41).

LC-MS/MS analysis was next employed to evaluate the effects of GAA on the metabolite profile and metabolic homeostasis of subcutaneous adipose tissue in Tibetan sheep. A total of 1,156 lipid metabolites were detected in both negative and positive ion modes, among which DG (16:0/20:4), DG (16:1/18:1), DG (16:0/18:2), TG (16:0/16:0/18:1), and Cer (d18:1/18:1) were identified as representative compounds. A strong positive correlation was found between Cer (d18:1/18:1) and MDA levels. In the GAA-treated groups, Cer (d18:1/18:1) levels remained stable while MDA content declined significantly, indicating attenuated oxidative damage in adipose tissue. This effect likely results from the upregulation of *IDNK* and *MAPKAPK5* (42), which strengthens antioxidant capacity, thereby partially suppressing lipid peroxidation and inhibiting the oxidative stress signals that promote Cer (d18:1/18:1) synthesis (43).

GAA treatment reduced DG (16:0/20:4) levels, with the most pronounced decrease in the 0.10% group. This reduction is primarily attributed to two reasons. First, downregulation of *PLA2G4A*, the rate-limiting enzyme for arachidonic acid (AA) release from membrane phospholipids (44), decreased free AA availability and thereby limited synthesis of the pro-inflammatory precursor DG (16:0/20:4). Second, upregulation of *PIK3CB* enhanced insulin signaling and lipogenic efficiency (45), promoting the conversion of DG (16:0/20:4) into triglycerides (TG) and reducing its accumulation in adipocytes (46). Consistent with this view, DG (16:1/18:1) and DG (16:0/18:2) levels also declined in the GAA groups, further indicating that enhanced insulin signaling facilitates diglyceride conversion. Collectively, the efficient channeling of DG into TG and the concomitant reduction of inflammatory mediators suggest that GAA not only improves lipid metabolism but also offers a rationale for optimizing fatty acid composition.

GAA treatment increased the TG (16:0/16:0/18:1) content. As a primary storage lipid, TG (16:0/16:0/18:1) serves as a stable energy reserve in adipocytes (47). The level of this TG species correlated positively with T-AOC, suggesting that insulin-driven anabolism maintains redox homeostasis. Sufficient NADPH maintained the antioxidant system, thereby enhancing the accumulation of TG (16:0/16:0/18:1) (48). Together with the stable levels of Cer (d18:1/18:1) and the decreased DG (16:0/20:4), these lipid profile changes indicate that GAA facilitates fatty acid metabolism while supporting insulin signaling integrity, adipose metabolic homeostasis, and the control of inflammatory responses.

## 5 Conclusion

In conclusion, dietary supplementation with 0.10% GAA significantly reduced subcutaneous fat thickness, enhanced antioxidant capacity, and improved the fatty acid profile in Tibetan sheep. Correlation analysis between DEGs and DAMs suggested that these improvements may be mediated through enhanced antioxidant capacity and insulin signaling, though the specific mechanisms require further investigation. These results demonstrate the potential of GAA as a feed additive to regulate subcutaneous fat metabolism in Tibetan sheep.

## Data Availability

The datasets presented in this study can be found in online repositories. The names of the repository/repositories and accession number(s) can be found below: https://www.ncbi.nlm.nih.gov/, PRJNA1413406 https://ngdc.cncb.ac.cn/, OMIX014583.

## Supplementary material

Supplementary Table 1 RNA Seq data analysis.

Supplementary Table 2 The RSDs of the QC samples.

Supplementary Table 3 The median CVs for each lipid class.
