# Supplementary material for: Effects of rumen-protected guanidinoacetic acid on fat deposition, antioxidant capacity, fatty acid profiles in subcutaneous adipose of Tibetan sheep: integrated RNA-seq and LC-MS/MS analysis
Source: Front Vet Sci. 2026 Aug 25;13:1866223. doi: 10.3389/fvets.2026.1866223 (PMC13552267; doi:10.3389/fvets.2026.1866223)
Supplement: Supplementary file 1 [file Table_1.docx]

| Supplementary Table 1 RNA-Seq data analysis | | | | | | | |
| --- | --- | --- | --- | --- | --- | --- | --- |
| Sample | Raw reads | Raw bases | Clean reads | Clean bases | Q20 | Q30 | GC pct |
| HG1 | 46503068 | 6.98G | 45046344 | 6.76 | 98.77 | 96.43 | 50.61 |
| HG2 | 48669532 | 7.3G | 46825370 | 7.02 | 98.73 | 96.33 | 50.78 |
| HG3 | 46319372 | 6.95G | 44785032 | 6.72 | 98.74 | 96.41 | 51.03 |
| HG4 | 47202418 | 7.08G | 46075238 | 6.91 | 97.67 | 93.49 | 50.84 |
| HG5 | 43395428 | 6.51G | 42245718 | 6.34 | 98.74 | 96.33 | 50.33 |
| HG6 | 44461428 | 6.67G | 43524538 | 6.53 | 98.71 | 96.27 | 49.64 |
| MG1 | 46892060 | 7.03G | 45714964 | 6.86 | 98.74 | 96.32 | 50.10 |
| MG2 | 47500194 | 7.13G | 46196074 | 6.93 | 98.77 | 96.36 | 50.79 |
| MG3 | 51781878 | 7.77G | 50135052 | 7.52 | 98.66 | 96.09 | 50.91 |
| MG4 | 47390344 | 7.11G | 46360448 | 6.95 | 98.9 | 96.73 | 46.25 |
| MG5 | 47611766 | 7.14G | 46344524 | 6.95 | 98.75 | 96.39 | 49.48 |
| MG6 | 48118252 | 7.22G | 45561504 | 6.83 | 98.73 | 96.36 | 49.12 |
| LG1 | 57138814 | 8.57G | 54174020 | 8.13 | 98.74 | 96.35 | 48.15 |
| LG2 | 40774478 | 6.12G | 39260864 | 5.89 | 98.61 | 95.88 | 50.04 |
| LG3 | 47542674 | 7.13G | 45954416 | 6.89 | 98.73 | 96.29 | 50.73 |
| LG4 | 43211440 | 6.48G | 41166152 | 6.17 | 98.69 | 96.23 | 50.49 |
| LG5 | 49793462 | 7.47G | 48671442 | 7.30 | 98.73 | 96.34 | 50.42 |
| LG6 | 41239300 | 6.19G | 40329422 | 6.05 | 98.51 | 95.86 | 50.65 |
| CON1 | 48127966 | 7.22G | 46632444 | 6.99 | 98.75 | 96.37 | 51.11 |
| CON2 | 45575360 | 6.84G | 44481240 | 6.67 | 98.74 | 96.34 | 50.80 |
| CON3 | 45484230 | 6.82G | 44150700 | 6.62 | 98.76 | 96.37 | 50.43 |
| CON4 | 48561514 | 7.28G | 47029006 | 7.05 | 98.72 | 96.3 | 50.86 |
| CON5 | 46928028 | 7.04G | 45411108 | 6.81 | 98.9 | 96.79 | 50.65 |
| CON6 | 54148514 | 8.12G | 52652520 | 7.90 | 98.78 | 96.47 | 50.44 |

Note: HG1, HG2, HG3, HG4, HG5, and HG6 represent the six biological replicates of the HG group; MG1 through MG6 represent the six biological replicates of the MG group; LG1 through LG6 represent the six biological replicates of the LG group; and CON1 through CON6 represent the six biological replicates of the control group.
